# Insufficient Resolution Response in the Hippocampus of a Senescence-Accelerated Mouse Model — SAMP8

**DOI:** 10.1007/s12031-014-0346-z

**Published:** 2014-06-10

**Authors:** Xiuzhe Wang, Elena Puerta, Angel Cedazo-Minguez, Erik Hjorth, Marianne Schultzberg

**Affiliations:** 1 Department of Neurobiology, Care Sciences and Society, Section of Neurodegeneration, Karolinska Institutet, 141 86 Stockholm, Sweden; 2 Department of Neurobiology, Care Sciences and Society, Section of Alzheimer’s Disease Research Center, Karolinska Institutet, Stockholm, Sweden; 3 Department of Pharmacology, School of Pharmacy, University of Navarra, Pamplona, Spain

**Keywords:** Aging, Alzheimer, Lipoxygenase, LXA_4_, Resolution of inflammation, RvD1, Tau

## Abstract

Aging is the primary risk factor for Alzheimer’s disease (AD), and it is known that inflammation is associated with both aging and AD. To resolve inflammation, biosynthesis of the specialized pro-resolving mediators (SPMs) is enhanced in a programmed and active manner. We investigated the effect of age on resolution by analyzing hippocampal tissue from 2- and 9-month-old senescence-accelerated mouse prone 8 (SAMP8), as well as age-matched senescence-accelerated mouse resistant 1 (SAMR1). Pro-inflammatory markers increased upon age in SAMP8 mice and were also higher than those in age-matched SAMR1 mice. However, neither SPMs nor their receptors were enhanced upon age in SAMP8 mice compared to age-matched SAMR1 mice. Analysis of SPM biosynthetic enzymes revealed elevated levels of leukocyte type 12-lipoxygenase (L12-LOX) and decreased 5-LOX levels upon age in SAMR1 mice, whereas they remained unchanged in SAMP8 mice. Moreover, we found partial co-localization of L12-LOX and amyloid beta (Aβ) staining, as well as correlation between L12-LOX and phosphorylated tau levels in SAMP8, but not SAMR1 mice. Thus, we conclude that the resolution response in SAMP8 mice is insufficient to counteract the increased inflammation with age, and this may have a role in the development of AD-like pathologies.

## Introduction

Increased proportion of aged individuals is a global phenomenon, raising concerns about age-related diseases, including Alzheimer’s disease (AD). AD is the most common type of dementia and a progressive neurodegenerative disease with no cure up to date. The etiology of AD is still not clear, despite the fact that the two pathological hallmarks, increased senile plaques consisting of amyloid β (Aβ) and neurofibrillary tangles composed of hyperphosphorylated tau (P-tau), have been known for over 100 years. While genetic factors that promote disease development have been identified, age is the primary risk factor for AD (Kawas et al. 2000).

Inflammation is known to be increased in aging, as indicated by increased levels of serum pro-inflammatory cytokines, such as interleukin (IL)-6 and tumor necrosis factor-α (TNF-α) (Wei et al. 1992; de Gonzalo-Calvo et al. 2010), and activation of glial cells in the brain (Ogura et al. 1994; Sheffield and Berman 1998; Campuzano et al. 2009). In AD, the age-related changes are even more pronounced (McGeer et al. 1987; Carpenter et al. 1993; Swardfager et al. 2010; Serrano-Pozo et al. 2011). There is ample evidence suggesting the pathogenic role of inflammation in AD. Long-term consumption of nonsteroidal anti-inflammatory drugs (NSAIDs) was shown to associate with lower prevalence of AD (McGeer et al. 1990; Stewart et al. 1997, in´t Veld et al. 2001). In AD animal models, by ablating or blocking p40, a subunit of the pro-inflammatory cytokines IL-12 and IL-23, AD-like pathologies and cognitive impairment were reduced (Vom Berg et al. 2012). Moreover, inflammation has been shown to regulate the processing of amyloid precursor protein (APP) and, thus, influences Aβ production (Sastre et al. 2008). Hence, it is reasonable to suspect a role of inflammation in the pathogenesis of AD. The exaggerated increase of inflammation in the AD brain suggests that AD could be a result of abnormal aging of the brain (Herrup 2010).

It is not until recent years that it became clear that inflammation is balanced by a programmed active process termed resolution (Serhan 2010), in which activation of inflammatory cells is reduced, levels of pro-inflammatory cytokines are downregulated, anti-inflammatory cytokines are upregulated, the tissue is healed, and homeostasis is restored (Serhan 2011). Resolution is mediated by specialized pro-resolving mediators (SPMs), including lipoxins (LXs), resolvins (Rvs), protectins/neuroprotectins (PDs/NPDs), and maresins (MaRs) (Serhan 2010). Biosynthesis of SPMs involves sequential oxidization of polyunsaturated fatty acids (PUFAs) by lipoxygenases (LOXs) and/or cyclooxygenase (COX), including leukocyte type 12-LOX (L12-LOX, mouse homologue to human 15-LOX-1) and 5-LOX (Recchiuti and Serhan 2012). In a skin inflammation model, the increased biosynthesis of LXA_4_ in the inflammation site shortly after the peak of pro-inflammatory activities demonstrates a typical programmed resolution response to inflammation (Levy et al. 2001). By activating their receptors, SPMs initiate and promote resolution of inflammation (Serhan et al. 2011; Bannenberg and Serhan 2010). The LXA_4_ receptor/formyl peptide receptor 2 (ALX/FPR2, also named as FPR2, ALX, or FPR2/ALX) is the receptor for both LXA_4_ and RvD1 (Fiore et al. 1994; Krishnamoorthy et al. 2012), and the chemokine receptor 23 (ChemR23) is the receptor for RvE1 (Arita et al. 2005) and a chemoattractant protein, chemerin (Wittamer et al. 2003). Both of these SPM receptors are G protein-coupled receptors.

Failure of resolution may lead to chronic inflammation and continuous tissue destruction, which may finally cause tissue dysfunction (Serhan 2007). Since both AD and “healthy aging” without dementia have signs of elevated inflammation, a dividing line could be an impaired resolution response specific for AD. To date, it is not known how resolution of inflammation in the brain is affected by aging. In the present study, we have analyzed the major markers for resolution in 2- and 9-month-old senescence-accelerated mouse prone 8 (SAMP8), a mouse model for accelerated aging with AD-like pathology and symptoms (Pallas et al. 2008). Age-matched animals of the senescence-accelerated mouse resistant 1 (SAMR1), which have a relatively normal aging process with intact cognitive function, were used as controls. SAMP8 and SAMR1 mice are selected by phenotype difference from one common background strain, AKR/J mice (Takeda et al. 1981; Miyamoto et al. 1986). By comparing these two different aging models, we tested the hypothesis that an abnormal aging process related to AD displays insufficiency in the mechanisms governing resolution of inflammation.

## Materials and Methods

### Animals

Male SAMP8 and SAMR1 mice were purchased from Harlan Laboratories (Barcelona, Spain). The mice were housed in a humidity- and temperature-controlled environment with a 12:12-h light–dark cycle. The animals had access to food and water ad libitum*.* The usage of animals in this study was approved by the Ethics Committee of the University of Navarra.

Brain tissues from 2- and 9-month-old SAMP8 and SAMR1 mice were analyzed with regard to factors involved in the resolution of inflammation by enzyme immunoassay (EIA), western blotting, and immunohistochemistry. Animals used for the biochemical assays were euthanized by intraperitoneal (i.p.) injection of a lethal dose of pentobarbital sodium (150 mg/kg body weight). The brains were dissected immediately, and the hippocampi from the left and right hemispheres were taken for EIA and western blotting, respectively. The tissues were kept at -80 °C until further analysis. Animals employed for morphology were anesthetized with i.p. injection of pentobarbital sodium (50 mg/kg body weight) and perfused with 4 % paraformaldehyde (PF) solution through the left ventricle. The brains were dissected immediately after perfusion, post-fixed in 4 % PF at 4 °C overnight, and subsequently soaked in 10 % sucrose at 4 °C until further processing.

### Enzyme Immunoassay

The LXA_4_ enzyme immunoassay (EIA) kit (Oxford Biochemical Research, MI, USA) and the resolvin D1 (RvD1) EIA kit (Cayman Chemical, Ann Arbor, USA) were used according to the manufacturers’ instructions for the analysis of extracts of the hippocampus from 2- and 9-month-old SAMP8 mice (both *n* = 5), 2-month-old SAMR1 mice (*n* = 4), and 9-month-old SAMR1 mice (*n* = 5). Briefly, the hippocampus from the left hemisphere was homogenized in ethanol and centrifuged at 1,500 *g* for 15 min. The supernatants were diluted by UltraPure water (Cayman Chemical, Ann Arbor, USA) and acidified to final pH 3.5. The acidified samples were added to a methanol preconditioned C18 column (Waters Corporation, MA, USA), and the column was subsequently washed by water and hexane. The SPMs were eluted by adding methyl formate to the column. Eluted solution was evaporated by nitrogen gas, and finally the extracted samples were resuspended by the extraction buffer supplied with the LXA_4_ EIA kit.

### Western Blotting

The hippocampus from the right hemisphere (*n* = 5 animals per group) was homogenized by sonication in a homogenization buffer containing 20 mM Tris–HCl, pH 6.8, 137 mM NaCl, 2 mM EDTA, 0.5 mM IBMX, 2 nM okadaic acid, 1 % protease inhibitor cocktail (Sigma-Aldrich Co., Saint Louis, USA), and phosphatase inhibitors (Halt^TM^ cocktail, Pierce-Bradford, IL, USA). The homogenates were centrifuged at 20,000 *g* for 15 min at 4 °C, and the supernatants collected for analysis. Protein determination was performed by a bicinchoninic acid kit (Sigma-Aldrich Co., Saint Louis, USA). Samples containing 40 μg protein each were mixed with an equal volume of 2× Laemmli buffer (Sigma-Aldrich Co., Saint Louis, USA) and boiled at 95 °C for 5 min, after which the samples were electrophoresed in 10 % SDS-polyacrylamide gel and transferred to a 4.5-μm nitrocellulose membrane. After blocking in 5 % nonfat milk for 45 min at room temperature (RT), the membranes were incubated at 4 °C overnight with the following primary antibodies raised in rabbit against: L12-LOX (1:1,000), 5-LOX (1:600) (Cayman Chemical, Ann Arbor, USA), ChemR23 (1:500), and ALX/FPR2 (1:500) (Santa Cruz, CA, USA). After washing, the membranes were incubated with horseradish peroxidase (HRP)-conjugated secondary antibodies for 2 h at RT. Finally, the membranes were developed by ECL reagent (GE health Care, Buckinghamshire, UK), and the signals detected by a CCD camera (Fuji Film, Tokyo, Japan). Analysis of the bands was carried out using the MultiGauge software (Fuji Film, Tokyo, Japan).

### Immunohistochemistry

The fixed brains (*n* = 4 per group except *n* = 5 for the 9-month-old SAMP8 group) were sectioned at 12 μm thickness in a cryostat (Leica Microsystems, IL, USA) and mounted onto polarized glass slides. The sections were kept at −20 °C until further analysis. For immunofluorescent staining, the sections were blocked for 30 min at RT with 5 % normal serum and then incubated overnight at 4 °C with primary antibodies as follows: rat anti-F4/80 1:100 (AbD Serotec, Puchheim, Germany); rabbit anti-ALX/FPR2 1:100 (Santa Cruz, CA, USA); and rabbit anti-ChemR23 1:200, anti-L12-LOX 1:200, and anti-5-LOX 1:200 (all from Cayman Chemical, Ann Arbor, USA). After washing, the slides were incubated with the appropriate secondary antibodies for 1 h at RT, washed, and then mounted in fluorescence mounting medium (Dako, Stockholm, Sweden).

Double labeling was performed on sections incubated with a mixture of (i) one of the above primary antibodies, and the neuronal marker, mouse anti-NeuN 1:500 (Chemicon/Millipore, Billerica, MA, USA); (ii) primary antibodies to 5-LOX and antibodies for the astrocyte marker rat anti-glial fibrillary acidic protein (GFAP, 1:400; Invitrogen, Carlsbad, CA, USA), or the microglial marker rat anti-F4/80 (1:100; AbD Serotec, Puchheim, Germany); (iii) rabbit anti-L12-LOX 1:200 and mouse anti-Aβ 4G8 1:100 (Signet/Covance, Dedham, MA, USA). A mixture of the appropriate secondary antibodies was used to detect the staining. Analysis was carried out under a fluorescence microscope (Nikon Eclipse 800, Tokyo, Japan).

### Statistical Analysis

Statistical analysis was performed with SPSS software (version 21.0, IBM Corporation, NY, USA). The univariate general linear model (GLM) was used, considering strain and age as two dependent variables. *p* values less than 0.05 were considered as statistically significant. All statistical data are expressed as mean ± standard error of the mean (SEM).

## Results

### Levels of Pro-resolving and Pro-inflammatory Markers

**Fig. 1:**
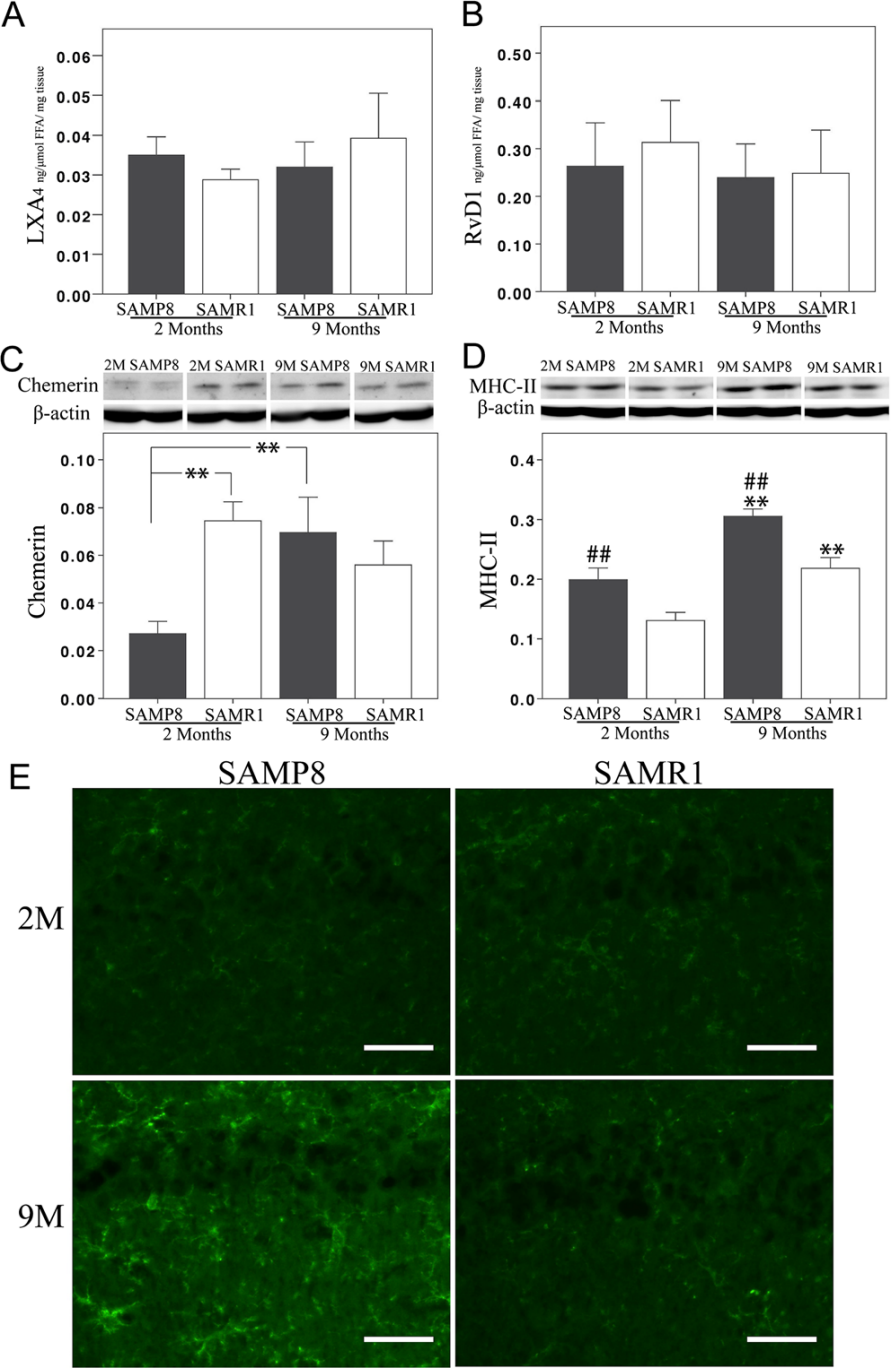
**a**–**e** SPMs and pro-inflammatory markers in the hippocampus of SAMP8 and SAMR1 mice. **a**, **b** Unchanged levels of LXA_4_ and RvD1 in SAMP8 mice compared to age-matched controls, and in both strains upon aging, as assessed by EIA. **c** Western blot data show lower levels of chemerin in SAMP8 mice at 2 months of age, but an increase with age reaching the same level as in SAMR1 mice at 9 months; **p < 0.01. **d** The MHC-II levels are higher in SAMP8 mice at both 2 and 9 months of age (##*p* < 0.01, SAMP8 vs. age-matched SAMR1), and the SAMR1 mice show a decrease with age (**p < 0.01, 9 vs. 2 months). **e** A markedly stronger microglial staining for F4/80 is seen in SAMP8 mice compared to SAMR1 mice at 9 months, as well as an increase with age in SAMP8 mice. *Scale bar* = 40 μm. Data are presented as mean ± SEM

Pro-resolving and pro-inflammatory profiles were analyzed in the hippocampus of SAMP8 and SAMR1 mice. Levels of LXA_4_ and RvD1 were not different between SAMP8 and age-matched SAMR1 mice, nor between 2- and 9-month-old mice in either strain (Fig. 1a, b).

The pro-inflammatory profile was assessed by analysis of chemerin, MHC-II, and F4/80. Levels of chemerin, a chemoattractant protein with pro-inflammatory effects, were higher in 9- than in 2-month-old SAMP8 mice. In the SAMR1 strain, there was a trend, albeit not statistically significant, toward lower levels of chemerin at 9 months (Fig. 1c). Higher levels of MHC-II were found in the hippocampus of both 2- and 9-month-old SAMP8 mice as compared to age-matched SAMR1 (Fig. 1d). In addition, the MHC-II levels were higher in 9- than in 2-month-old SAMP8 mice (Fig. 1d). Analysis of F4/80, a marker for activated microglia, showed similar staining in SAMP8 and SAMR1 mice at 2 months of age, whereas at 9 months of age, a higher staining intensity was observed in SAMP8 mice as compared to age-matched SAMR1 mice (Fig. 1e).

### SPM Receptors

**Fig. 2:**
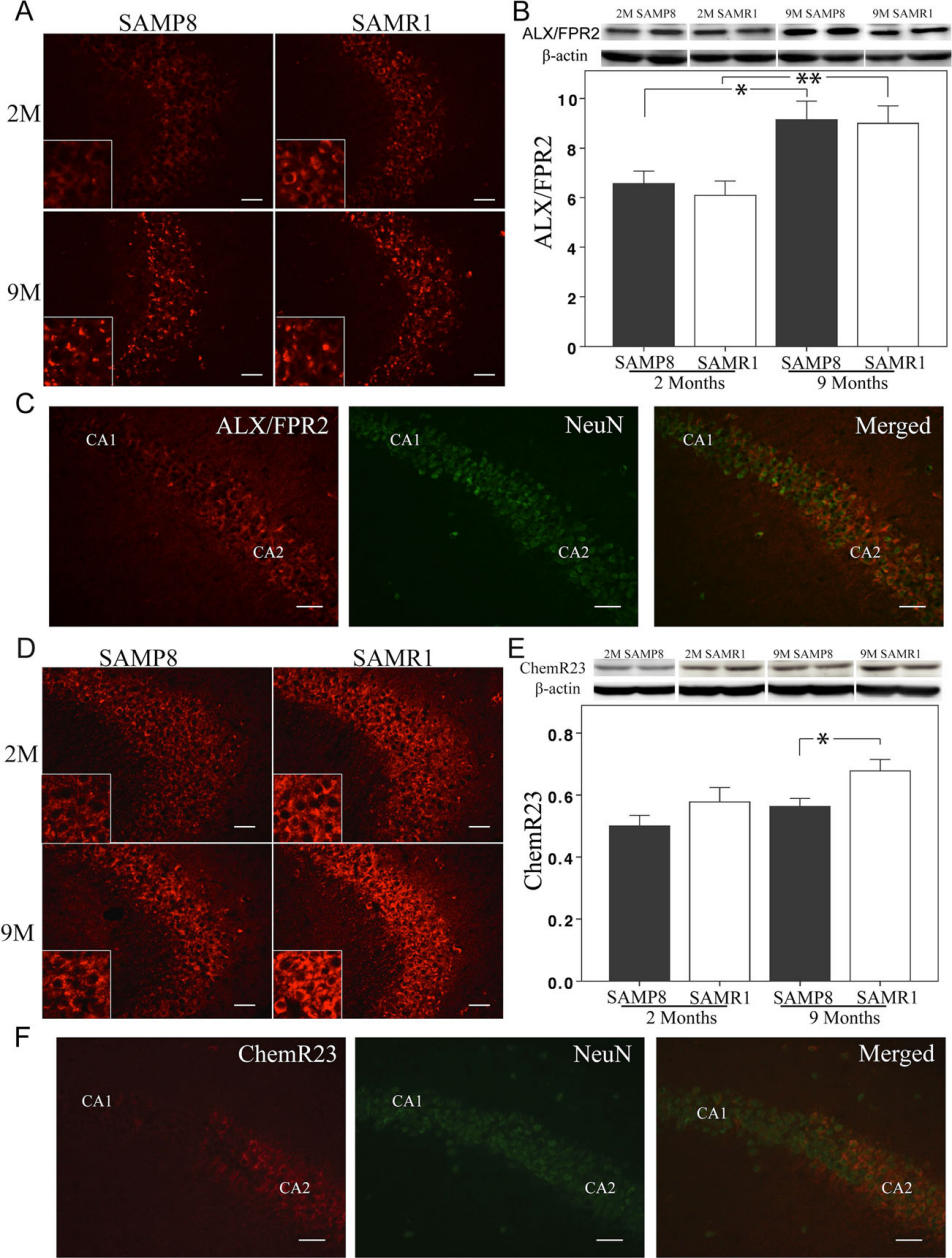
**a**–**f** Analysis of ALX/FPR2, the receptor for LXA_4_ and RvD1, and ChemR23, the receptor for RvE1, in SAMP8 and SAMR1 mice. **a** Immunofluorescence staining for ALX/FPR2 in pyramidal cells in the CA2-3 regions. **b** Data from western blot analysis of the hippocampus show higher levels of ALX/FPR2 at 9 months of age in both strains, but no difference between the SAMP8 and SAMR1 mice. **c** Double staining shows localization of ALX/FPR2 in NeuN-positive pyramidal neurons in the CA2 region. The ALX/FPR2 labeling is markedly weaker in CA1 than in CA2. **d** Immunofluorescence staining of ChemR23 in pyramidal cells in the CA2-3 regions. **e** Data from western blot analysis of the hippocampus show lower levels of ChemR23 in SAMP8 mice compared to SAMR1 at 9 months. There is no difference with age in either strain. **f** Double staining shows localization of ChemR23 in NeuN-positive pyramidal neurons in the CA2 region. No staining for ChemR23 can be seen in CA1. *Scale bar* = 40 μm. Data are presented as mean ± SEM. *p < 0.05, **p < 0.01

In view of unchanged LXA_4_ and RvD1 levels during aging in SAMP8 mice, we next investigated if there was a change in their receptors. Immunohistochemical analysis showed staining of ALX/FPR2 in pyramidal cells in the hippocampus of both SAMP8 and SAMR1 mice (Fig. 2a, c). The neuronal staining was considerably weaker in cornu ammonis area 1 (CA1) than in CA2-4 (Fig. 2c). There was an increase with age in both SAMP8 and SAMR1 mice, but no difference between the two mouse strains, as shown both by immunohistochemistry and western blot (Fig. 2a, b).

Analysis of ChemR23, the receptor for RvE1 and chemerin, showed a similar distribution as that for ALX/FPR2 (Fig. 2d, f), i.e., staining of pyramidal cells, and with higher intensity in CA2-4. At 9 months of age, the hippocampal levels of ChemR23 were lower in SAMP8 than in SAMR1 mice, whereas no difference could be seen between the young and older mice in either strain (Fig. 2e).

### Enzymes Involved in SPM Biosynthesis

**Fig. 3:**
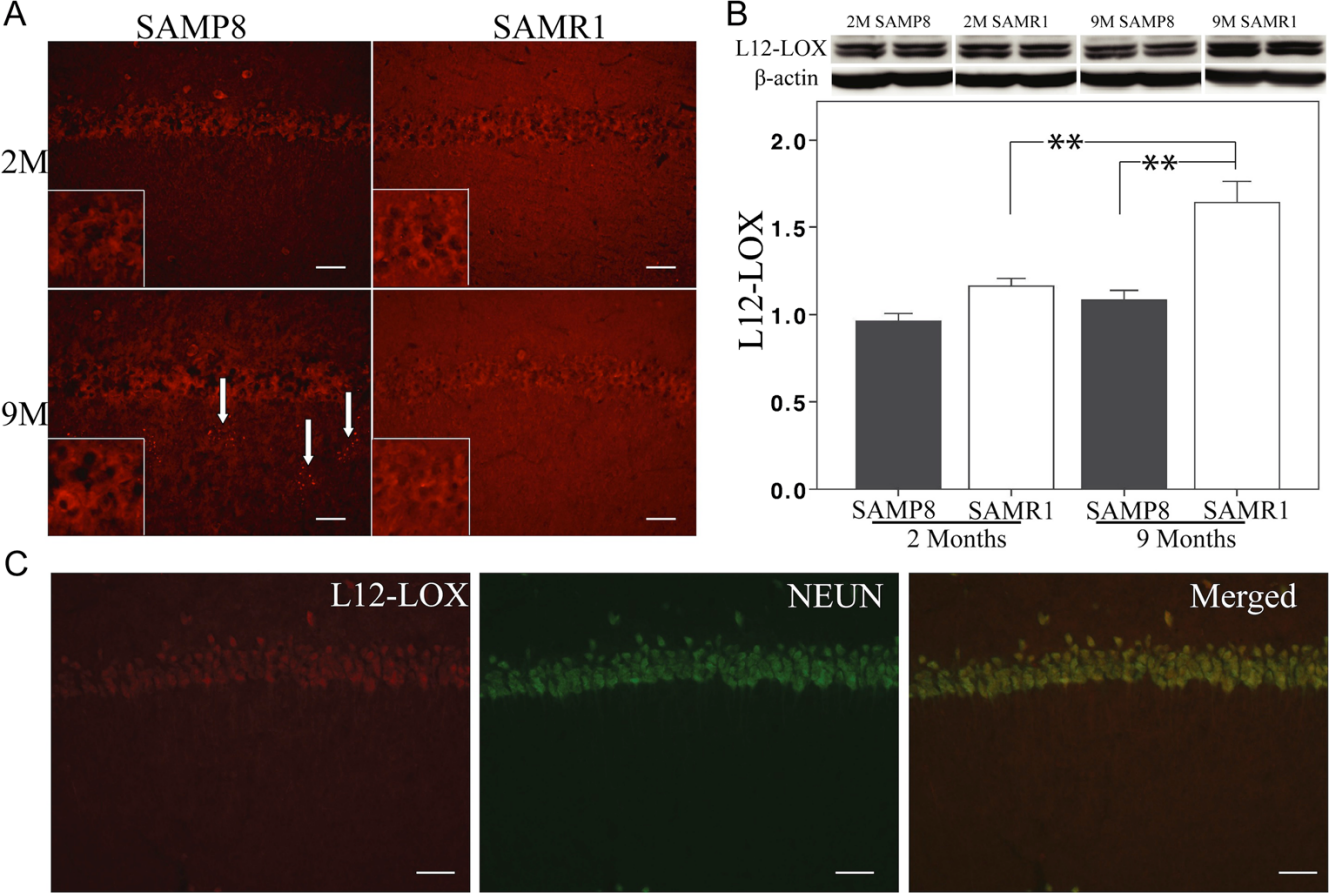
**a**–**c** Analysis of L12-LOX. **a** Immunofluorescence staining of L12-LOX in pyramidal cells. In 9-month-old SAMP8 mice, clusters of punctate staining can be seen in the hippocampus (*arrows*). **b** Data from western blot analysis of the hippocampus show lower levels of L12-LOX in SAMP8 mice compared to SAMR1 mice at 9 months. There is no difference with age in the SAMP8 strain, but an increase in the SAMR1 strain. **c** Double staining shows localization of L12-LOX in NeuN-positive pyramidal neurons. *Scale bar* = 40 μm. Data are presented as mean ± SEM. **p < 0.01

L12-LOX is the first enzyme to catalyze arachidonic acid (AA) or docosahexaenoic acid (DHA) during the production of LXA_4_ (Serhan et al. 1986) or RvD1 (Sun et al. 2007). It is functionally and structurally similar to 15-LOX-1 and sometimes named as 12/15-LOX together with 15-LOX-1 (L12-LOX is used through out the current study) (Haeggstrom and Funk 2011). Immunohistochemical analysis showed staining of L12-LOX in pyramidal neurons (Fig. 3a, c) throughout CA1 to CA4. Interestingly, clusters of punctate immunofluorescence for L12-LOX were observed in the hippocampus of 9-month-old SAMP8 mice, but not in anyone of the other animal groups (Figs. 3a and 5a). Western blot analysis demonstrated lower levels of L12-LOX in SAMP8 compared to SAMR1 mice at 9 months (Fig. 3b). Aging did not affect the L12-LOX levels in SAMP8 mice, but an increase with age was seen in the SAMR1 strain.

**Fig. 4:**
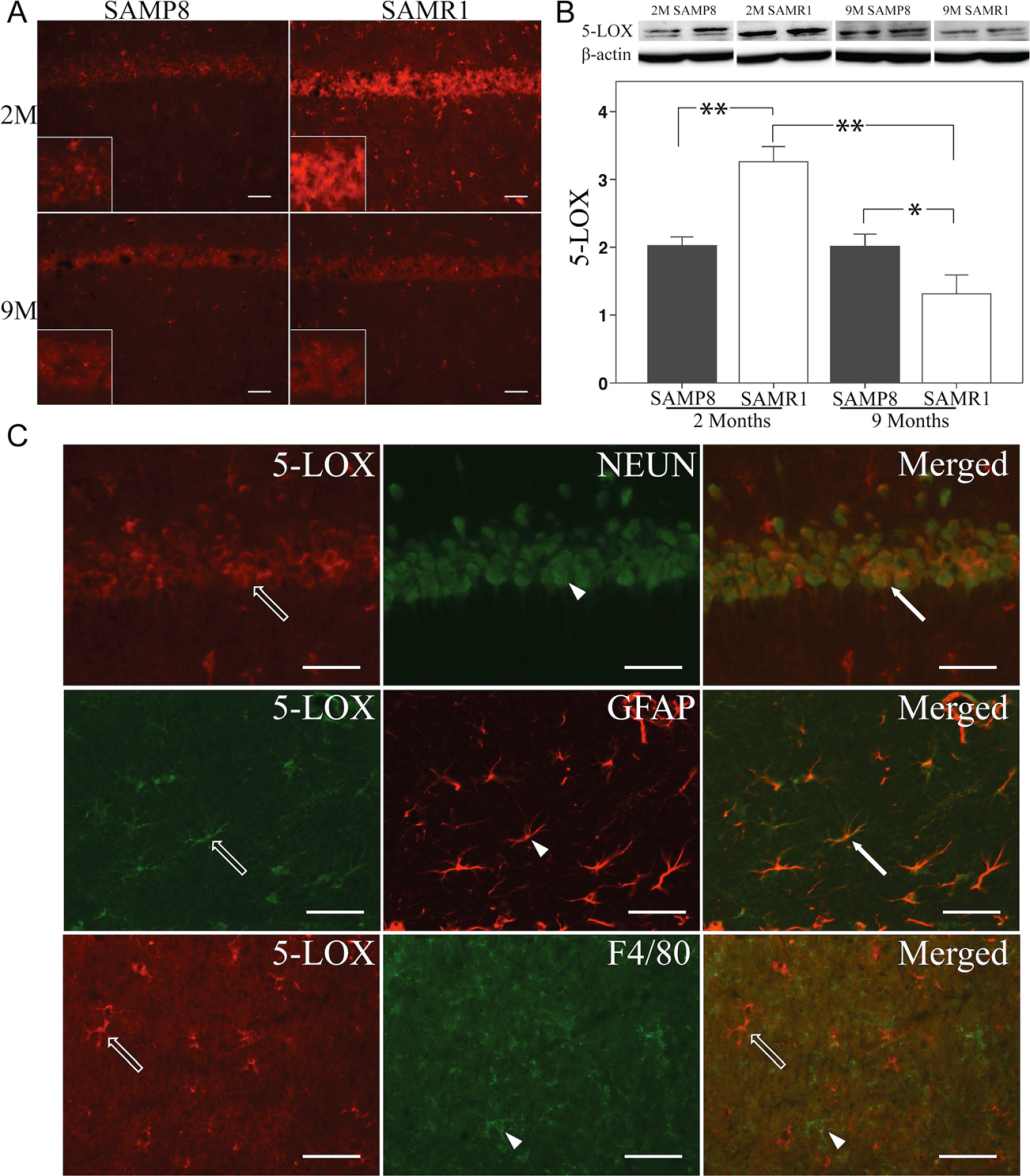
**a**–**c** Analysis of 5-LOX. **a** 5-LOX is seen in both pyramidal neurons and glia. **b** Data from western blot analysis of the hippocampus show lower levels of 5-LOX in 2-month-old SAMP8 mice and higher levels in 9-month-old SAMP8 mice, compared to age-matched SAMR1 mice. There is no difference with age in the SAMP8 strain, but a decrease in the SAMR1 strain. **c** Double staining shows localization of 5-LOX in NeuN-positive pyramidal neurons (*arrows and arrowhead in top panel indicate cell with colocalization*), as well as in GFAP-positive astrocytes (*arrows and arrowhead in middle panel indicate cell with colocalization*), but not in F4/80-positive microglia (*arrows and arrowhead in lower panel indicate separate cells*). *Scale bar* = 40 μm. Data are presented as mean ± SEM. *p < 0.05, **p < 0.01

5-LOX is the second enzyme involved in the production of LXA_4_ (Serhan et al. 1986) and RvD1 (Sun et al. 2007). In the hippocampus of both SAMP8 and SAMR1 mice, 5-LOX immunostaining was found in neurons and glial cells (Fig. 4a). As shown by double immunohistochemistry with GFAP, 5-LOX staining can be seen in both pyramidal neurons and astrocytes (Fig. 4c). There was no co-localization with the microglial marker F4/80 (Fig. 4c). The overall change in 5-LOX levels was assessed by western blot. The levels were lower in 2-month-old and higher at 9-month-old SAMP8 compared to age-matched SAMR1 mice (Fig. 4b). SAMP8 mice showed no change in 5-LOX levels with age. In contrast, a significant decrease in 5-LOX was found in 9-month-old SAMR1 compared with 2-month-old SAMR1 mice (Fig. 4b).

### AD-Related Markers

**Fig. 5:**
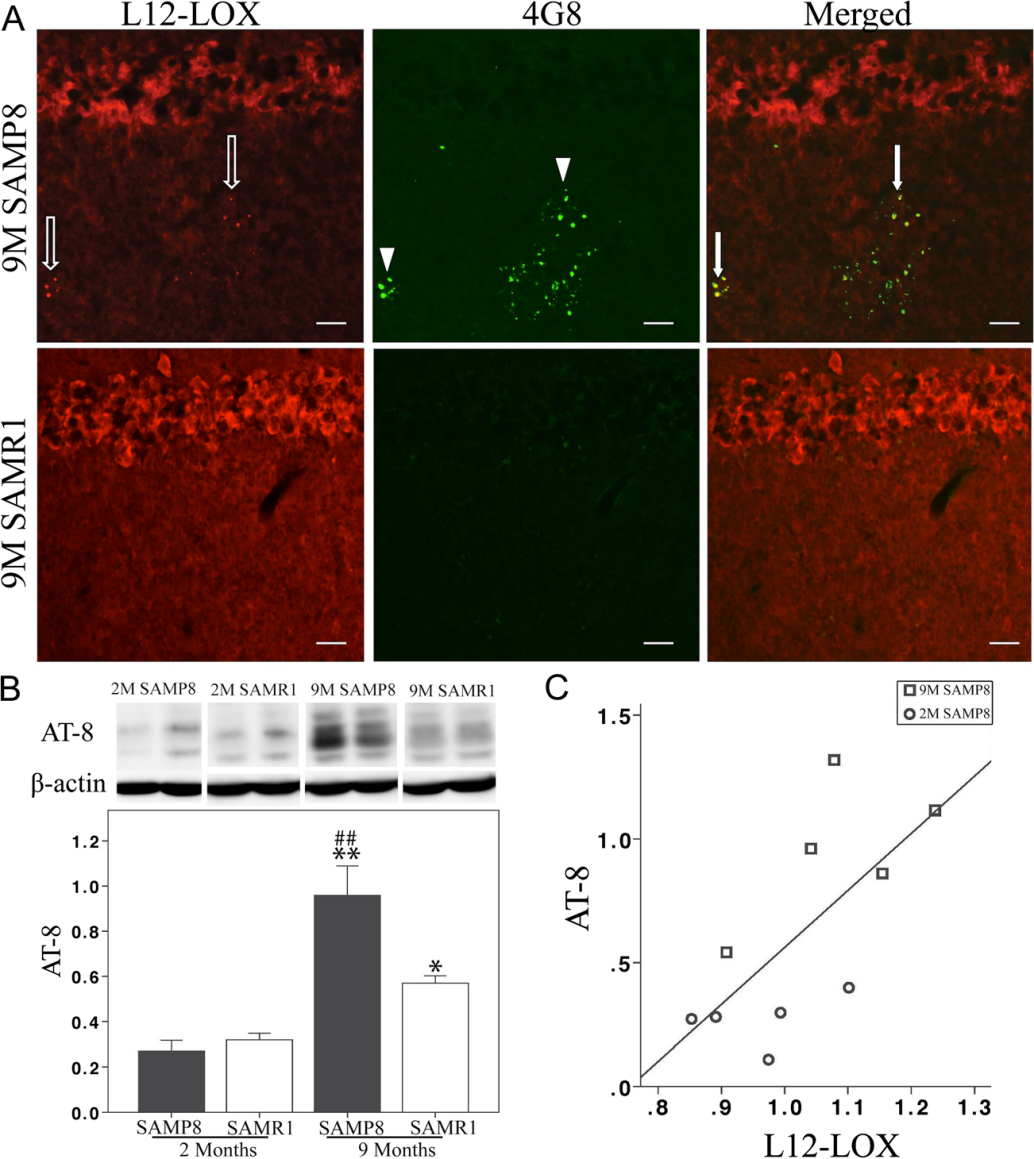
**a**–**c** Analysis of L12-LOX in relation to Aβ and P-tau in SAMP8 and SAMR1 mice. **a** Double staining shows the co-localization (*arrows*) of L12-LOX with Aβ (*arrowheads*) (using the 4G8 antibody) in clusters of punctate structures in the hippocampus of 9-month-old SAMP8 mice. **b** Levels of AT-8 P-tau are higher in 9-month-old SAMP8 than in age-matched SAMR1 mice. Data from western blot analysis of the hippocampus show an increase with age in P-tau levels in both strains. *Bar* = 40 μm. **c** Positive correlation between the levels of L12-LOX and AT-8 P-tau (Pearson correlation test, *r* = 0.679, *p* < 0.05). Data are presented as mean ± SEM. ##*p* < 0.01, SAMP8 vs. age-matched SAMR1; **p < 0.01, 9 vs. 2 months

Analysis of L12-LOX revealed clustered immunostaining in the group of 9-month-old SAMP8 mice, but not in any other group (Fig. 3a). The staining pattern is similar to that described previously for Aβ deposits in aging SAMP8 mice (Del Valle et al. 2010; Manich et al. 2011). Double immunostaining with 4G8 antibodies (also used in Del Valle et al. 2010; Manich et al. 2011) revealed partial co-localization of L12-LOX and Aβ in the hippocampus of 9-month-old SAMP8 mice (Fig. 5a).

The levels of P-tau were analyzed by western blot using AT-8 antibodies (Ser202/Thr205 p-tau). Hippocampal P-tau levels were significantly higher in 9-month-old SAMP8 compared to SAMR1 mice of the same age (Fig. 5b). There was an increase with age in both strains (Fig. 5b). Correlation analysis between AT-8 and L12-LOX showed a positive correlation (Pearson correlation test, *r* = 0.679, *p* < 0.05) in the SAMP8 strain (Fig. 5c), but not in the SAMR1 strain.

## Discussion

Resolution of inflammation mediated by SPMs emerges as an important factor in various inflammation-associated diseases, including AD. In previous studies, we have demonstrated lower levels of the pro-resolving factor LXA_4_ in cerebrospinal fluid (CSF) samples from AD patients, as well as higher levels of SPM receptors and a biosynthetic enzyme, in the AD hippocampus (Wang et al. 2014). In the present study, by using SAMP8 and SAMR1 mice, we investigated how the resolution pathway is regulated in aging, the primary risk factor for AD.

**Fig. 6:**
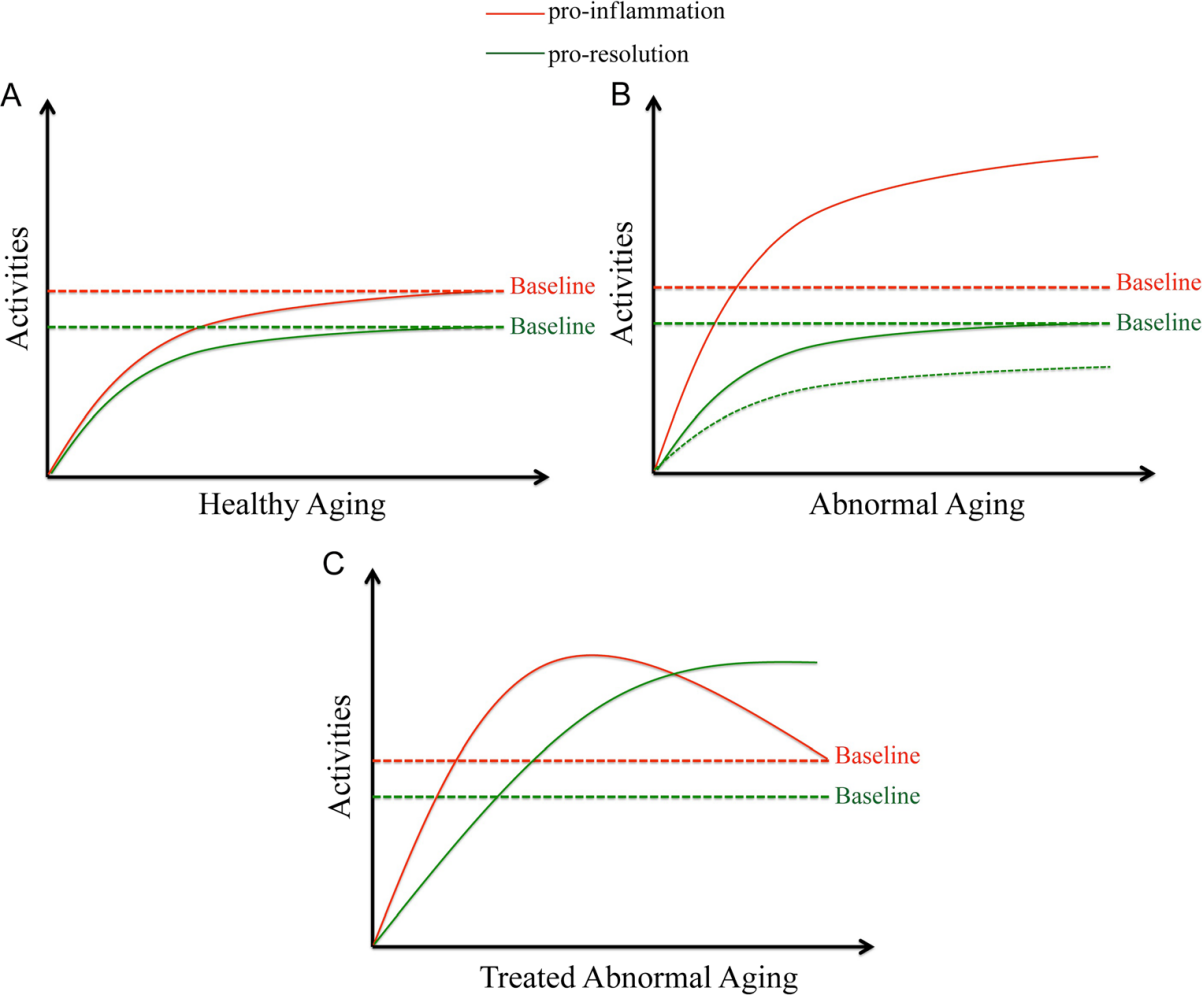
**a**–**c** Schematic representations of the hypothesis for resolution in healthy aging, abnormal aging related to AD, and treatment for abnormal aging, as concluded from the data obtained in SAMP8 and SAMR1 mice. **a** Healthy aging (baselines for inflammation and resolution) is characterized by a small increase in pro-inflammatory activities, accompanied by increased pro-resolving activities (due to increased SPM receptor levels). **b** Abnormal aging related to AD develops excessive pro-inflammatory activities as compared to baseline (i.e., upon aging in SAMR1 mice), but the pro-resolving activities are insufficient and remain at baseline levels that meet only the baseline inflammation. The *dashed curve* indicates that pro-resolving activities may be even lower than baseline, due to increased levels of Aβ, binding to ALX/FPR2. **c** Treatment including supplement of SPMs or modulation of LOXs may resolve the excessive inflammation in abnormal aging

Healthy aging is associated with low-grade inflammation and is sometimes referred to as “inflamm-aging” (Franceschi et al. 2000). In a previous study, IL-1β levels were elevated with age in the SAMR1 strain (Tha et al. 2000). In agreement with this, we found a slight increase associated with aging in the microglial activation marker MHC-II in SAMR1 mice. This was accompanied by unchanged levels of the SPMs LXA_4_ and RvD1, whereas ALX/FPR2, the receptor for LXA_4_ and RvD1, was upregulated with age. These data demonstrate the regulation of pro-resolving signaling during a healthy aging progress (see schematic Fig. 6). Moreover, since L12-LOX and 5-LOX are both needed to sequentially oxidize AA or DHA to produce LXA_4_ or RvD1, it is hypothesized that the stable levels of LXA_4_ and RvD1 with age in SAMR1 mice may be a consequence of increased L12-LOX levels, but decreased levels of 5-LOX.

The SAMP8 strain is a model of accelerated aging, obtained by selecting for this phenotype from the same background strain as SAMR1 (Takeda et al. 1981; Miyamoto et al. 1986). Thus, without being transgenic for human AD mutations, SAMP8 mice display impaired learning and memory and increased Aβ levels and phosphorylation of tau with age (Pallas et al. 2008). In the present study, SAMP8 mice showed a more marked increase in inflammation upon aging as compared to normal aging SAMR1 mice. This was shown by higher levels of MHC-II and F4/80 in aged SAMP8 mice, confirming previous studies showing increased levels of the pro-inflammatory cytokines IL-1β, IL-6, and TNF-α (Tha et al. 2000; Liu et al. 2013). Furthermore, the levels of chemerin were dramatically increased upon age in SAMP8 mice, whereas no significant change was seen in SAMR1 mice. Chemerin has been proposed as a pro-inflammatory marker in multiple sclerosis, due to its function as a chemoattractant for inflammatory cells (Lande et al. 2008; Graham et al. 2009). There was no significant change in the levels of its receptor ChemR23, but the resulting elevated chemerin/ChemR23 axis in abnormal aging lends further evidence for a state of exaggerated inflammation.

The resolution response regulates inflammation by an active process mediated by SPMs (Recchiuti and Serhan 2012), and the excessive inflammation in the aged SAMP8 mice may require a stronger pro-resolving signaling by SPMs compared to the baseline in SAMR1 mice. However, there was no difference in the levels of LXA_4_ and RvD1 between the aged SAMP8 and the aged SAMR1 mice, even though the inflammation was more pronounced in SAMP8 mice. The levels of the receptor ALX/FPR2 were elevated upon aging in SAMP8 mice, but were not different from the levels in age-matched SAMR1 mice. This indicates that the resolution axis, i.e., LXA_4_/RvD1–ALX/FPR2, remained at a baseline level similar to SAMR1 mice. Thus, the resolution in SAMP8 mice could be considered unresponsive to the inflammation. It may be hypothesized that the cause of this unresponsiveness is the increased AD-like molecular pathology present in SAMP8 mice. Notably, Aβ levels increase with age in SAMP8 mice (Takemura et al. 1993; Del Valle et al. 2010; Manich et al. 2011; and our own data), and this neurotoxic peptide also binds to ALX/FPR2 (Le et al. 2001; Yazawa et al. 2001). As a result, the net effect of competition between increased Aβ and unchanged LXA_4_ and RvD1 may be decreased pro-resolving signaling and increased harmful signal transduction through ALX/FPR2 (see schematic Fig. 6b).

The LOX enzymes govern the biosynthesis of SPMs, but they also have other distinctive functions, complicating the interpretation of their role. For example, elevated levels of L12-LOX in response to skin inflammation were associated with enhanced LXA_4_ biosynthesis (Levy et al. 2001), and overexpression of L12-LOX was beneficial to a rabbit periodontitis model due to more production of LXA_4_ (Serhan et al. 2003). However, overexpression of L12-LOX has also been shown to increase Aβ production (Chu et al. 2012) and enhanced tau-phosphorylation at Ser202/Thr205 (AT-8) (Giannopoulos et al. 2013) in Tg2576 APP transgenic mice. Here we show co-localization of L12-LOX with the Aβ clusters found in aged SAMP8 mice (Del Valle et al. 2010; Manich et al. 2011), indicating a potential novel association between L12-LOX and Aβ. We also found a positive correlation between L12-LOX and tau-phosphorylation at Ser202/Thr205 in SAMP8, but not in SAMR1 mice. These data may indicate a detrimental role of L12-LOX in aged SAMP8 mice. However, there were higher L12-LOX levels also in the aged SAMR1 mice, but without correlation with AD-like pathology. The paradox of L12-LOX function found in the present study indicates the need for further studies to characterize the regulation of L12-LOX function, e.g., regarding differential phosphorylation (Khanna et al. 2005). Notably, in addition to increased L12-LOX, we found that 5-LOX was decreased with age in SAMR1 mice. The same counter-regulation of L12-LOX and 5-LOX has been reported in IL-4-treated dendritic cells and was proposed as a mechanism mediating immune modulation and anti-inflammatory effects (Spanbroek et al. 2001). In the abnormal aging SAMP8 mice, this counter-regulation did not happen, and the two LOXs remained at unchanged levels with age. This absence of counter-regulation of L12-LOX and 5-LOX may contribute to dysfunction of resolution in SAMP8 mice.

In summary, we provided data on how the resolution pathway in the brain is regulated to meet inflammation during normal aging using an animal model. Moreover, during abnormal aging, accompanied by AD-like pathologies, there was insufficient resolution response to resolve the excessive inflammation compared to normal aging. This insufficiency may be partially due to dysregulation of LOXs, and their role during development of AD-like pathologies needs further characterization. Future studies will be required to establish whether supplementary treatment with SPMs or modulating LOXs may ameliorate aging-related AD pathologies by re-establishing the balance between resolution and inflammation (see schematic Fig. 6c).
